# Total arch and descending thoracic aortic replacement for massive hemoptysis requiring CPR caused by intrapulmonary penetration of chronic dissecting aortic aneurysm: a case report

**DOI:** 10.1186/s40792-022-01573-9

**Published:** 2022-12-15

**Authors:** Tsubasa Mikami, Takashi Yamauchi, Satoshi Sakakibara, Yoshito Ito, Hitoshi Suhara, Yukio Hayashi, Toru Kuratani, Takafumi Masai, Yoshiki Sawa

**Affiliations:** 1 Department of Cardiovascular Surgery, Sakurabashi Watanabe Hospital, 2-4-32, Umeda, Kita-Ku, Osaka, Osaka 530-0001 Japan; 2 Department of Cardiovascular Surgery, Osaka University Graduate School of Medicine, Suita, Osaka Japan; 3 Department of Cardiovascular Surgery, Higashiosaka City Medical Center, Higashiosaka, Osaka Japan; 4 Anesthesiology Service, Sakurabashi Watanabe Hospital, Osaka, Osaka Japan

**Keywords:** Chronic dissecting aortic aneurysm, Aortopulmonary fistula, Straight incision with a rib-cross approach

## Abstract

**Background:**

Intrapulmonary penetration of the thoracic aorta is a rare, life-threatening complication of a chronic dissecting aortic aneurysm. It causes massive hemoptysis requiring prompt intervention to prevent fatal airway bleeding. A surgical approach that enables diverse surgical maneuvers and intraoperative organ protection is crucial.

**Case presentation:**

A 62-year-old man, who underwent graft replacement of the ascending aorta for an acute type A aortic dissection 20 months before, developed massive hemoptysis and cardiac arrest. The hemoptysis was secondary to an aortopulmonary fistula from a rapidly expanding dissecting aortic aneurysm. However, a successful return of spontaneous circulation was achieved with cardiopulmonary resuscitation, including establishment of veno-arterial extracorporeal membrane oxygenation. The patient successfully underwent a total arch and descending thoracic aortic replacement. This was achieved by a median sternotomy combined with a left thoracotomy using a straight incision with a rib-cross (SIRC) approach. The patient was uneventfully discharged and remained well for the following 2 years.

**Conclusions:**

When performing a surgical graft replacement for an aortopulmonary fistula with a thoracic aortic aneurysm, the surgical approach chosen is critical. A surgical procedure using a median sternotomy combined with a left thoracotomy and a SIRC approach can be an effective therapeutic option.

## Background

A thoracic aortic aneurysm (TAA) with intrapulmonary penetration is a type of aortopulmonary fistula (APF) [1, 2]. It can result in massive hemoptysis and is associated with high mortality despite modern, multidisciplinary treatments [1, 2]. Life-saving surgical intervention is difficult if preoperative massive airway bleeding cannot be promptly managed [3]. Moreover, surgery for a TAA-associated APF, particularly when performing graft replacement (GR), requires a wide range of surgical techniques. Therefore, a surgical approach that can secure a sufficient surgical field is essential. Here, we describe the emergency surgical approach required to manage a patient with massive hemoptysis from a TAA-associated APF.

## Case presentation

**Fig. 1:**
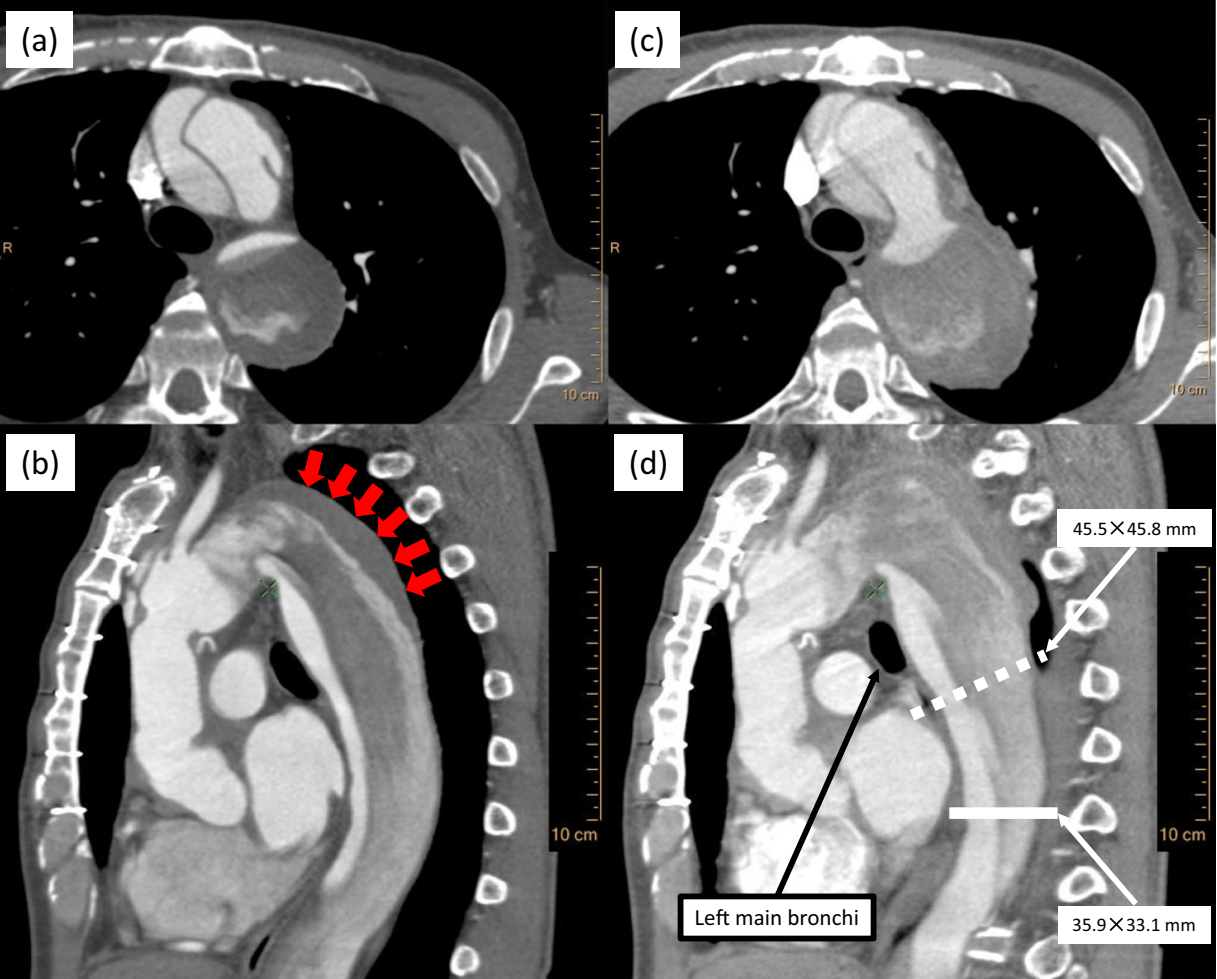
Preoperative computed tomography. The distal aortic arch expanded rapidly within 2 months; at 2 months before admission (**a** axial, **b** sagittal), thrombi are seen in the false lumen of the distal aortic arch (red arrows), and at admission (**c** axial, **d** sagittal). **d** White dotted line indicates the estimated distal anastomosis site, while the white solid line defines, where the distal anastomosis was actually performed

A 62-year-old man, who developed an acute type A aortic dissection and underwent GR of the ascending aorta (J-Graft 24 mm; Japan Lifeline, Tokyo, Japan) 20 months before, presented with a relatively fast-growing distal aortic arch enlargement during a routine follow-up visit and was scheduled for admission followed by surgical treatment. However, he presented with a high fever while awaiting hospitalization and was admitted urgently to our hospital. On admission, his body temperature was elevated at 38.2 °C. The patient’s laboratory test results revealed the following: white blood cell count was within the normal range at 8.6 × 10^9^/L, hemoglobin level was reduced at 6.5 mmol/L, serum creatinine level was within the normal range at 69.0 μmol/L, and C-reactive protein level was elevated at 115.4 mg/L. Transthoracic echocardiography indicated preserved cardiac function without vegetation. Computed tomography (CT) revealed a residual aortic dissection from the distal end of the vascular graft to the abdominal aorta; within 2 months, the maximum diameter of the distal aortic arch had rapidly expanded from 51 mm (Fig. 1a, b) to 64 mm (Fig. 1c, d). CT also confirmed a heterogeneous contrast effect in and around the false lumen of the dissecting distal aortic arch (Fig. 1c, d) without apparent systemic infection. We diagnosed a rapidly expanding dissecting aortic aneurysm (DAA) with the possibility of infection, and repeat surgery was scheduled urgently. However, the patient suddenly developed massive hemoptysis on the day preceding surgery and lost consciousness, resulting in cardiac arrest. Cardiopulmonary resuscitation (CPR) was immediately initiated, and percutaneous veno-arterial (VA) extracorporeal membrane oxygenation (ECMO) was established via the femoral vessels; the return of spontaneous circulation was confirmed. Hemoptysis, which persisted during CPR, was controlled by combining VA-ECMO and mechanical ventilation with a high level (15 cm H_2_O) of positive end-expiratory pressure (PEEP). Thereafter, the patient’s hemodynamics stabilized, and he gradually regained consciousness without any apparent neurological dysfunction. The DAA was believed to have perforated the lungs; therefore, he was transferred to the operating room for emergency surgery.

**Fig. 2:**
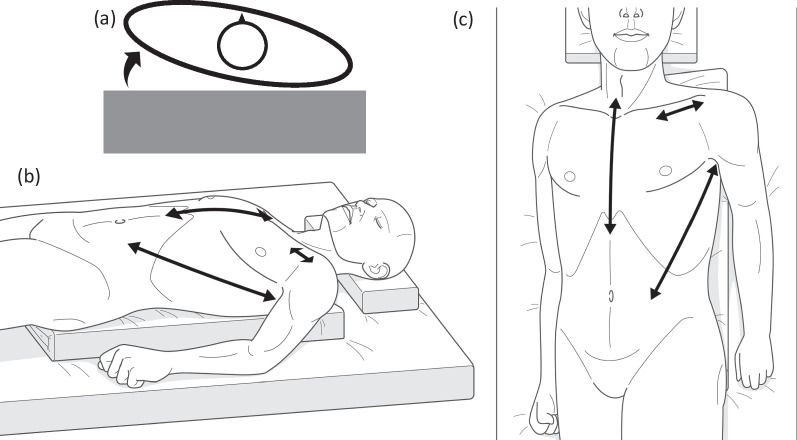
Schema of the patient’s intraoperative position and surgical incisions. Schema of (**a**) the patient’s intraoperative position from the head and (**b**, **c**) the surgical skin incisions of the median sternotomy, left thoracotomy, and left subclavian artery (for exposure of the left axillary artery)

Based on the preoperative CT scan, in addition to total arch replacement from the vascular graft anastomosed in the previous surgery, it was necessary, at least, to perform graft replacement of the proximal descending aorta (Fig. 1d). We simultaneously considered the possibility of the involvement of infection and the necessity for reliable hemostasis against hemoptysis. Therefore, preoperatively, we placed the patient in a slightly right lateral decubitus position (Fig. 2a) and performed a median sternotomy combined with a left thoracotomy using a straight incision with a rib-cross (SIRC) approach [4] (Fig. 2b, c) to manage the strong adhesions in the mediastinum and the left thoracic cavity; establish intraoperative organ protection, particularly for the myocardium and the brain; and secure a sufficient surgical field required for the extended thoracic aortic surgery. In addition to the division of the fourth to sixth ribs by the SIRC approach, an additional division from the eighth to tenth costal cartilages enabled us to secure an adequate surgical field despite strong adhesions in the thoracic cavity. The left lung was firmly adhered to the aorta, from the aortic arch to the descending aorta, and could not be separated from the proximal descending aorta. Meanwhile, the adhesion between the distal descending aorta and the left lung was relatively mild, which led to the decision of performing total arch and descending thoracic aortic replacement. The distal aortic arch was markedly dilated, and the bifurcation of the left subclavian artery was relatively deep; therefore, we decided to bypass to left axillary artery (AxA), anastomosed a vascular graft (J-Graft 9 mm; Japan Lifeline, Tokyo, Japan) to the left AxA, and temporarily clamped the proximal side of the vascular graft. Thereafter, we established an extracorporeal circuit (ECC) by incorporating this vascular graft as one of the arterial lines into the VA-ECMO system, in addition to the superior vena cava drainage and left ventricular venting.

Subsequently, we commenced gradual core cooling. Following the establishment of hypothermic circulatory arrest (urinary bladder temperature: 25.0 °C) and selective antegrade cerebral perfusion using the vascular graft anastomosed to the left AxA, we removed the flaps in the distal aortic arch, the thrombi in the false lumen, and any tissues with suspected infections. During this procedure, a 3 × 3 cm area of the lung parenchyma and pulmonary vessels were unexpectedly exposed. The area was believed to be related to the APF, which we directly closed as far as possible; there were no apparent signs of infection, including abscess formation. Although we successfully removed the APF, due to the inevitable iatrogenic lung injury, there was concern about the de novo development of extrapulmonary or airway hemorrhages. Therefore, we exposed the upper, middle, and lower branches of the pulmonary artery in the left hilar region in case ligation of the pulmonary arteries was required to control any hemorrhaging. After clamping the descending aorta at the T9 level, we resumed femoral artery perfusion (2 L/min) and ligated the intercostal arteries. Subsequently, we anastomosed a four-branched vascular graft (J-Graft 24 mm; Japan Lifeline, Tokyo, Japan) to the descending aorta using an open distal anastomosis technique while temporarily stopping femoral artery perfusion. Thereafter we started rewarming the patient. After a proximal anastomosis to the vascular graft of the ascending aorta and the reconstruction of the brachiocephalic artery, left carotid artery, and left AxA were performed, we started weaning the patient off the ECC.

As expected, a large amount of airway bleeding was confirmed in the left lung’s tracheal tube. Therefore, we ligated the upper branch of the pulmonary artery while observing the inside of the trachea using a bronchoscope. This temporarily reduced the bleeding; however, as airway bleeding recurred, ligation of the middle branch of the pulmonary artery was performed to suppress it. Thereafter, the patient’s hemodynamics and oxygenation were stabilized without VA or veno-venous ECMO support, and the operation was completed.

**Fig. 3:**
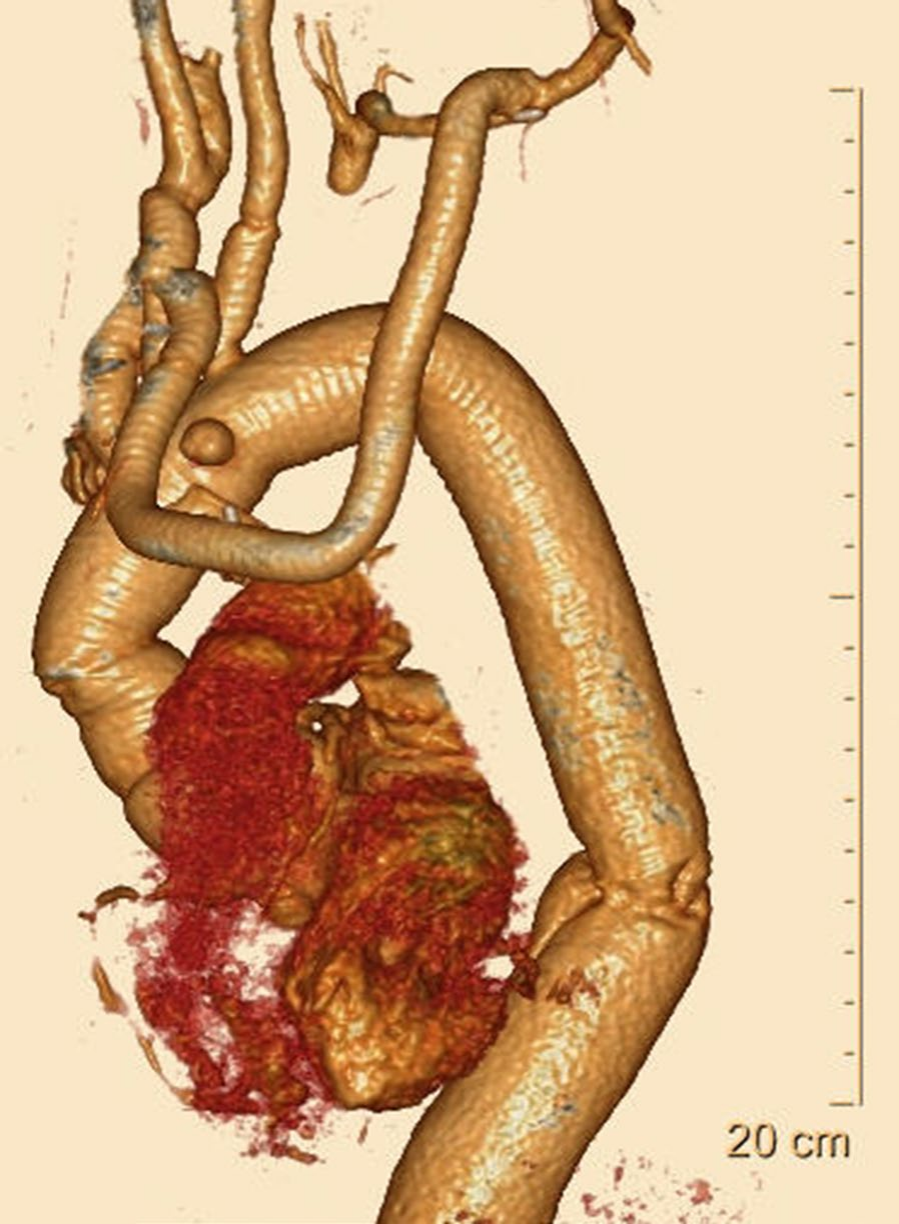
Postoperative three-dimensional computed tomography. The dissecting aortic arch aneurysm was completely excluded

The patient was extubated on postoperative day 1. There was no infection, which was confirmed by the negative results of the preoperative blood cultures and the tissues collected during surgery. The patient developed mediastinitis 5 weeks postoperatively, but omentopexy combined with antibiotic therapies sufficiently controlled the condition. The patient was eventually discharged 16 weeks postoperatively without any neurological or respiratory dysfunction. Postoperative 3D CT showed that the aortic arch aneurysm was completely resected (Fig. 3). The patient remained in good health for the following 2 years.

## Discussion

One of the critical factors to rescue patients with a TAA-associated APF is the preoperative control of the patient’s hemodynamic condition. In this case, managing the airway with a high level of PEEP combined with VA-ECMO might have contributed to reducing airway bleeding. We speculate that the rapidly expanding DAA could be attributed to the turbulence caused by abundant antegrade and retrograde blood flow into the false lumen (Fig. 1b) rather than infection.

In the surgical treatment of a TAA-associated APF, surgical GR or endovascular treatment with a stent graft (SG) is determined individually [1]. Surgical GRs can treat aneurysms and fistulas of the lungs or bronchi; however, large surgical invasion and high mortality are major issues [2]. On the contrary, endovascular treatment with an SG can be performed with minimal invasiveness and a reasonable survival rate [2]. However, this treatment may be inadequate due to the recurrence of the APF or postoperative SG infection [2]. In this case, we performed extended aortic GR for two reasons: first, the rapid return of spontaneous circulation after VA-ECMO establishment, without apparent neurological dysfunction, suggested that the patient would be able to tolerate the invasiveness of the surgical intervention. Second, there was the possibility of infection, which required adequate treatment.

The surgical approach to manage a TAA-associated APF needs to be carefully considered to secure a sufficient surgical field required for GR, because strong adhesions are likely to exist in the mediastinum and thoracic cavity, and it is necessary to cover a wide area from the ascending to descending aorta depending on the replacement range. Moreover, to prevent postoperative complications associated with high mortality, it is essential to establish adequate organ protection during surgery. Extensive aortic GR is a highly invasive surgery, although a left thoracotomy with median sternotomy can be an effective therapeutic option [1].

In this case, we had to perform, at least, total arch and proximal descending aortic replacement, based on the preoperative CT scan. Since the proximal descending aorta had enlarged to about 46 mm (Fig. 1d), an elaborate circumferential approximation of the dissected aortic wall and distal anastomosis of the vascular prosthesis were likely to be required in the deep mediastinum. Simultaneously, there was a possibility of replacing the descending aorta more distal than expected, depending on the spread of the infection. Furthermore, we should have considered the effect of adhesions from the previous surgery, the importance of intraoperative organ protection and the possibility of dealing with the pulmonary parenchyma and pulmonary vessels associated with the massive preoperative hemoptysis. Therefore, considering our proficiency in surgical techniques for emergency surgery, we selected a surgical approach that combined a left thoracotomy with median sternotomy. This enabled us to perform with firm confidence the administration of cardioplegia, left ventricle unloading, and reliable brain protection with selective antegrade cerebral perfusion even after the patient had undergone median sternotomy. We specifically selected the SIRC approach for the left thoracotomy [4], making it possible to perform surgical maneuvers from the distal aortic arch to the descending aorta and control airway bleeding in the same surgical field. The bleeding occurred during weaning the patient off the ECC and originated from the iatrogenic fistula between the pulmonary arteries and bronchioli. It was safely treated by selective and stepwise ligation of the pulmonary arteries. However, our approach has the potential for the distal aortic arch to be far from both median sternotomy and left thoracotomy because of the patient’s position. This may be the reason why the lung parenchyma and pulmonary vessels were unexpectedly exposed, and both the intercostal and bronchial arteries were particularly challenging to be dealt with. In addition, although the patient had not developed a lung abscess or respiratory dysfunction postoperatively, pulmonary resection would be generally preferred, considering the harmful effects of unresected lung whose feeding arteries were ligated as well as the resulting VQ mismatch. Moreover, simultaneous omentopexy should have been considered not only to control air leakage from the fistula but also to prevent graft infection, because the massive APF has a substantial risk of graft infection.

For extended aortic GRs, clamshell incision [5, 6] or anterolateral thoracotomy with partial sternotomy [7, 8] may also be feasible options. The former, also called bilateral anterior thoracotomy, is an approach commonly used in lung transplantation. Although favorable postoperative outcomes of this approach in extended aortic GR have been reported, the high postoperative respiratory complications are a concern [6]. In this case, it was difficult for us to choose this approach, because the patient had hemoptysis preoperatively and was likely to develop postoperative respiratory complications. The latter is an approach in which left thoracotomy is added to partial median sternotomy. Excellent postoperative outcomes of this approach in extended aortic GR have been reported [7, 8]. With this approach, it is possible to secure a wide surgical field from the aortic root to the distal descending aorta. Moreover, it is extremely useful for administering cardioplegia and establishing antegrade selective cerebral perfusion, and is comparable to median sternotomy in performing such procedures [8]. Therefore, it was considered to be an ideal surgical approach that met almost all of our requirements for the patient. However, we had insufficient experience using this method, and therefore, decided against it in this particular emergency case.

## Conclusions

We report a case of massive hemoptysis requiring CPR from intrapulmonary penetration of a chronic DAA. This was successfully treated with a prompt response to airway bleeding and extended thoracic aortic replacement. When performing surgical GR for a TAA-associated APF, the choice of surgical approach is critical; median sternotomy combined with left thoracotomy and an SIRC approach can be an effective therapeutic option.

## Data Availability

The data sets supporting the conclusions of this article are included within the article and its additional files.

## Abbreviations

TAA: Thoracic aortic aneurysm
APF: Aortopulmonary fistula
GR: Graft replacement
CT: Computed tomography
DAA: Dissecting aortic aneurysm
CPR: Cardiopulmonary resuscitation
VA: Veno-arterial
ECMO: Extracorporeal membrane oxygenation
PEEP: Positive end-expiratory pressure
SIRC: Straight incision with rib-cross
AxA: Axillary artery
ECC: Extracorporeal circuit
SG: Stent graft
